# Emergence of *Klebsiella pneumoniae* ST273 Carrying *bla*_NDM-7_ and ST656 Carrying *bla*_NDM-1_ in Manila, Philippines

**DOI:** 10.1089/mdr.2015.0205

**Published:** 2016-10-01

**Authors:** Andrew Chou, Marylette Roa, Michael A. Evangelista, Arielle Kae Sulit, Evelina Lagamayo, Brian C. Torres, David C. Klinzing, Maria Luisa G. Daroy, Josephine Navoa-Ng, Richard Sucgang, Lynn Zechiedrich

**Affiliations:** ^1^ Division of Infectious Diseases, Department of Medicine, Baylor College of Medicine, Houston, Texas.; ^2^ Department of Molecular Virology and Microbiology, Baylor College of Medicine, Houston, Texas.; ^3^ Research and Biotechnology Group, St. Luke's Medical Center, Quezon City, Philippines.; ^4^ Verna and Marrs McLean Department of Biochemistry and Molecular Biology, Baylor College of Medicine, Houston, Texas.; ^5^ Pathology Institute, St. Luke's Medical Center, Quezon City, Philippines.; ^6^ Tahoe Research Initiative, Incline Village, Nevada.; ^7^ Infection Control Service, St. Luke's Medical Center, Quezon City, Philippines.; ^8^ Department of Pharmacology, Baylor College of Medicine, Houston, Texas.

**Keywords:** metallo-beta-lactamase, carbapenemase, carbapenem resistant, molecular epidemiology, *Enterobacteriaceae*, whole genome sequencing

## Abstract

We sought to determine the epidemiology of carbapenem-resistant *Enterobacteriaceae* and to investigate the emergence of carbapenem-resistant *Klebsiella pneumoniae* in two teaching hospitals in Manila, Philippines. We screened 364 *Enterobacteriaceae* for carbapenem resistance between 2012 and 2013 and detected four carbapenem-resistant *K. pneumoniae* isolates from three different patients. We used whole genome sequencing to determine the antibiotic resistance profiles and confirmed the presence of carbapenemase genes by multiplex PCR. We used multilocus sequence typing and PCR-based replicon typing to genetically characterize the carbapenem-resistant isolates. The carbapenemase gene *bla*_NDM_ was detected in *K. pneumoniae* isolates from two patients. The first patient had ventilator-associated pneumonia and lumbar shunt infection from *K. pneumoniae* ST273 carrying *bla*_NDM-7_. The second patient had asymptomatic genitourinary colonization with *K. pneumoniae* ST656 carrying *bla*_NDM-1_. The third patient had a gluteal abscess with *K. pneumoniae* ST1 that did not carry a carbapenemase gene, but did carry *bla*_DHA-1_, *bla*_OXA-1_, and *bla*_SHV-1_. In this study, we report the first cases of *bla*_NDM_-carrying pathogens in the Philippines and add to the growing evidence of the worldwide spread of ST273 and NDM-7, a more efficient carbapenem hydrolyzer than NDM-1.

## Introduction

New-Delhi metallo-beta-lactamase-1 (NDM-1) is the most recently described metallo-beta-lactamase and has emerged as a global health threat.^1^ Similar to other metallo-beta-lactamases, NDM-1 can hydrolyze all beta-lactams except aztreonam. NDM-1 is a global health threat because the gene encoding NDM-1, *bla*_NDM-1_, is found on more diverse mobile genetic elements than other metallo-beta-lactamase genes.^2^ Since first detected in 2008, NDM-1 has been reported on every continent except the Antarctica, although the main reservoirs appear to be the Indian subcontinent, the Balkan region, and the Middle East.^3,4^ NDM-1 has been sporadically detected in Southeast Asia, but never in the Philippines, where previous regional surveillance of carbapenemases detected IMP-26 only.^5,6^ Of 24,684 isolates analyzed by the Philippines Department of Health, only 0.7% of *Klebsiella* were carbapenem resistant, although carbapenemase gene testing was not reported.^7^

During passive surveillance of antimicrobial resistance in two academic teaching hospitals, we identified the emergence of carbapenem-resistant *K. pneumoniae* and sought to determine which beta-lactamase and carbapenemase genes were present in these isolates. In this study, we report the detection of *bla*_NDM-1_ and *bla*_NDM-7_, which produces a more efficient carbapenem hydrolyzer than NDM-1, and the spread of *K. pneumoniae* ST273, a clone with outbreak and epidemic potential.

## Materials and Methods

### Microbial identification and antibiotic susceptibility testing

Collection of organisms was from January 2012 to February 2013 at two affiliated academic teaching hospitals in metropolitan Manila, Philippines. Organism identification and antibiotic minimum inhibitory concentrations (MICs) were determined by the VITEK-2 system using VITEK card AST-N261 (*bioMèrieux*, Paris, France) (Table 1). Carbapenemase activity was detected by the modified Hodge test. Microbiological tests were performed and interpreted according to the procedures of the Clinical Laboratory Standards Institute Performance Standards for Antimicrobial Susceptibility Testing: Twenty-second Informational Supplement M100-S21 (2011).

**Table 1.** MICs, Molecular Typing, and Antibiotic Resistance Genes of *Klebsiella pneumoniae* Isolates

	*Isolate, MIC (μg/ml)*			
*Antibiotic*	*ARPG-318*	*ARPG-379*	*ARP-664*	*ARPG-315*
Amikacin	4	≤2	≥64	32
Amoxicillin/clavulanate	≥32	≥32	≥32	≥32
Ampicillin	≥32	≥32	≥32	≥32
Cefepime	2	≥64	≥64	32
Cefoxitin	≤4	≥64	≥64	≥64
Ceftazidime	16	≥64	≥64	≥64
Ceftriaxone	≥64	≥64	≥64	≥64
Ciprofloxacin	≥4	≥4	≥4	≥4
Colistin	≤0.5	2	≥16	2
Ertapenem	≤0.5	≥8	≥8	≥8
Gentamicin	≥16	≥16	≥16	≥16
Imipenem	≤0.25	≥16	8	2
Meropenem	≤0.25	≥16	≥16	2
Piperacillin/tazobactam	32	≥128	≥128	≥128
Trimethoprim/sulfamethoxazole	≥320	≥320	≥320	≥320
Sequence type	ST147	ST273	ST656	ST1
Plasmid replicon type	Non-typeable	IncA/C	Non-typeable	Non-typeable
Beta-lactamase *bla* genes	CTX-M-15, OXA-1, SHV-11, TEM-1B	CTX-M-15, NDM-7, OXA-1, SHV-11, TEM-1B	CTX-M-15, NDM-1, OXA-1, SHV-1	DHA-1, OXA-1, SHV-1

ARPG-318 and ARPG-379 were sequential isolates collected from patient 1. ARP-664 was collected from patient 2. ARPG-315 was collected from patient 3.

MIC, minimum inhibitory concentration.

### Carbapenemase gene detection

Carbapenemase genes were amplified using a previously described multiplex PCR, PCR products were sequenced by the Sanger method (Lone Star Labs, Houston, TX), and DNA sequences were compared with those in GenBank and from the Lahey Clinic database of β-lactamases (http://lahey.org/studies/).^4,8,9^

### Multilocus sequence typing and PCR-based replicon typing

Multilocus sequence typing was performed using established protocols (http://bigsdb.web.pasteur.fr) with the following modifications: PCR was performed with 2.5 mM MgCl_2_ and the annealing temperature was 60°C. DNA sequences were searched against the Institut Pasteur MLST database. Plasmid replicon typing was performed using previously established multiplex PCRs with the following conditions: 2 μl of extracted DNA and 2 mM MgCl_2_ were used in each 25 μl reaction.^10^ Primers, other reagents, and the thermocycling settings were as previously described.^11^

### Whole genome sequencing

DNA was extracted with the QIAamp DNA Mini Kit (Qiagen, Hilden, Germany). Sequencing was performed on an Illumina MiSeq platform and underwent *de novo* assembly. Beta-lactamase genes were detected using ResFinder 2.1.^12^

## Results

### Detection of carbapenem-resistant *Enterobacteriaceae*

Three hundred sixty-four *Enterobacteriaceae* were collected, including 181 *Escherichia coli*, 135 *Klebsiella spp.*, 19 *Enterobacter spp.*, and 38 other *Enterobacteriaceae*. Four *Klebsiella pneumoniae* isolates from three unique patients were resistant to at least one carbapenem (ertapenem, imipenem, or meropenem). All carbapenem-resistant *K. pneumoniae* were detected during the same month and were investigated as a possible outbreak. One isolate (ARPG-315) displayed low-level meropenem resistance (MIC to meropenem of 2 μg/ml, imipenem of 2 μg/ml, ertapenem of ≥8 μg/ml). Three *K. pneumoniae* isolates (designated as ARPG-379, ARPG-383, and ARP-664) had high-level meropenem resistance (≥16 μg/ml).

### Clinical histories of patients with carbapenem-resistant *K. pneumoniae* infections

Patient 1 was a 70-year-old woman transferred to St. Luke's Medical Center Global City in 2013 because of a subarachnoid hemorrhage requiring intubation and placement of a lumbar drain. After transfer, on hospital day 3, she developed ventilator-associated pneumonia caused by a carbapenem-susceptible *K. pneumoniae* (ARPG-318) and was treated with meropenem. On day 33, the patient developed a lumbar shunt infection and ventilator-associated pneumonia from a carbapenem-resistant *K. pneumoniae* (ARPG-379 and ARPG-383). The patient's treatment was escalated to include colistin and amikacin, which resolved the infection. On day 66, the patient had an acute neurological decline and was discharged from the hospital in poor neurological condition at the family's request.

Patient 2 was a 92-year-old man admitted to St. Luke's Medical Center Quezon City in 2013 for a gastrostomy tube exchange. Postoperative course was complicated by respiratory failure and ventilator-associated pneumonia caused by *Pseudomonas aeruginosa*, which was treated with meropenem. On day 21, a urine culture grew carbapenem-resistant *K. pneumoniae* (ARP-664). The culture was interpreted as colonization, and no antimicrobial agent was administered. No active genitourinary infections occurred, but on day 46, the patient's respiratory function worsened and the patient died.

Patient 3 was a 23-year-old man admitted to St. Luke's Medical Center Global City in 2013 with acute lymphocytic leukemia and received induction chemotherapy, which was complicated by prolonged neutropenia and recurrent gluteal abscesses. The abscess cultures initially grew extended-spectrum beta-lactamases (ESBL) producing *E. coli*, but subsequently grew carbapenem-resistant *K. pneumoniae* (ARPG-315). The patient was treated by incision and drainage of the abscess, and was discharged home in good health after resolution of the neutropenia.

### Molecular typing of carbapenem-resistant *K. pneumoniae*

From patient 1, the carbapenem-susceptible *K. pneumoniae* isolate (ARPG-318) was typed as ST147 carrying *bla*_CTX-M-15_, *bla*_OXA-1_, *bla*_SHV-11_, and *bla*_TEM-1B_. PCR-based replicon typing did not detect a typeable plasmid. The lack of carbapenemase gene was confirmed by multiplex PCR. The carbapenem-resistant *K. pneumoniae* isolate ARPG-379 was identified as ST273. ARPG-379 had a positive modified Hodge test and carried *bla*_CTX-M-15_, *bla*_NDM-7_, *bla*_OXA-1_, *bla*_SHV-11_, and *bla*_TEM-1B_. The presence of *bla*_NDM-7_ was confirmed by multiplex PCR and the product sequences match a previously reported *bla*_NDM-7_ (GenBank Accession No. JX262694.1). PCR-based replicon typing detected the IncA/C type plasmid. From patient 2, the carbapenem-resistant *K. pneumoniae* isolate (ARP-664) was identified as ST656 and had a positive modified Hodge test. Carbapenemase gene sequences matched a previously reported *bla*_NDM-1_ (GenBank Accession No. FN396876.1) except for a C > T synonymous single-nucleotide polymorphism at position 564 (Table 1). PCR-based replicon typing did not detect a typeable plasmid. From patient 3, the carbapenem-resistant *K. pneumoniae* isolate (ARPG-315) was identified as ST1 and carried *bla*_DHA-1_, *bla*_OXA-1_, and *bla*_SHV-1_. No carbapenemase genes were detected by either whole genome sequencing or multiplex PCR.

## Discussion

To our knowledge, these are the first two cases of *K. pneumoniae* carrying *bla*_NDM_ in the Philippines and the first report of the spread of ST273 and ST656. This report shows that the two *K. pneumoniae* carrying *bla*_NDM_ are not epidemiologically linked; the isolates were genetically distinct. Each patient's isolate had different multilocus sequence types and different *bla*_NDM_ variants.

Previous surveillance of *Enterobacteriaceae* carrying genes encoding carbapenemases in the Philippines detected only *bla*_IMP-26_ from *K. pneumoniae* ST626 and ST903.^13^ *K. pneumoniae* ST273 was initially identified in Europe and has been reported in Italy, Norway, Russia, and the United Kingdom.^14–17^ These studies reported that ST273 isolates harbor various carbapenemase genes, including *bla*_KPC_, *bla*_NDM-1_, and *bla*_VIM_, and recognized ST273 as having high epidemic potential. *K. pneumoniae* ST273 encodes a single allelic variant compared to ST147, which caused a nosocomial outbreak of NDM-1-producing clone in Mainland China.^18,19^ *K. pneumoniae* ST656 is a recently reported sequence type and has only been reported in China carrying *bla*_CTX-M-14_.^20^ Little is known about ST656, and the isolate from patient 2 (ARP-664) is the first report of ST656 carrying a carbapenemase gene. The isolate from patient 3 did not carry any known carbapenemase gene, but did carry a plasmidic AmpC beta-lactamase gene and ESBL-encoding genes, which are known to contribute to the carbapenem resistance phenotype when coexpressed with porin modification or loss.^21^

Ours is the fourth report of *K. pneumoniae* carrying *bla*_NDM-7_, which encodes the carbapenemase NDM-7, a newly described variant of NDM of particular concern because NDM-7 has a greater hydrolytic activity against carbapenems than NDM-1. The prior reports of *K. pneumoniae* carrying *bla*_NDM-7_ were from two case reports of single patients and a report of an outbreak involving seven patients.^22–24^ There also have been six cases of infections with *bla*_NDM-7_-carrying *E. coli*.^8,22,25–27^ These isolates harboring *bla*_NDM-7_ have rapidly emerged in multiple continents since first being detected in 2012, and have been found in Germany, India, Japan, Spain, and the United States.^8^

This report contributes to the local and regional epidemiology of carbapenem-resistant *Enterobacteriaceae*. Tracking carbapenem-resistant *Enterobacteriaceae* and mechanisms of resistance are clinically significant because new antimicrobial agents, such as ceftazidime/avibactam, are not active against bacteria carrying metallo-beta-lactamase genes, such as *bla*_NDM_, but may be active against bacteria carrying *bla*_KPC_. Surveillance will provide guidance on the utility of new antimicrobial agents to treat multidrug-resistant gram-negative infections.^28^ Given the concerns of its high epidemic potential, *K. pneumoniae* ST273 and *bla*_NDM_ must be closely monitored and rapidly reported.
